# Somatic Mitochondrial DNA Point Mutations Used as Biomarkers to Demonstrate Genomic Heterogeneity in Primary Prostate Cancer

**DOI:** 10.1155/2020/7673684

**Published:** 2020-08-28

**Authors:** Christian Arstad, Kristin Taskén, Paulo Refinetti, Ulrika Axcrona, Karl-Erik Giercksky, Per Olaf Ekstrøm

**Affiliations:** ^1^Department of Tumor Biology, Institute for Cancer Research, The Norwegian Radium Hospital, Oslo University Hospital, Oslo, Norway; ^2^Chaire de Statistique Appliquee, Section de Mathematiques, EPFL, Lausanne, Switzerland; ^3^Department of Pathology, The Norwegian Radium Hospital, Oslo University Hospital, Oslo, Norway

## Abstract

Primary prostate tumor heterogeneity is poorly understood, leaving research efforts with challenges regarding the initiation and advancement of the disease. The growth of tumor cells is accompanied by mutations in nuclear and in mitochondrial genomes. Thus, mitochondrial DNA mutations may be used as tumor cell markers. By the use of laser capture microdissection coupled with assays for mitochondrial point mutation detection, mtDNA mutations were used to trace mutated cells at a histological level. Point mutations in mtDNA were determined in 12 primary prostate cancers. The tumors represent different pathology-prognostic grade groups. Known mutational hotspots of the mtDNA were scanned for heteroplasmy. All specimens with mtDNA heteroplasmy were subsequently subsampled by laser capture microdissection. From a total number of 1728 microsamples, mitochondrial DNA target sequences were amplified and base substitutions detected by cycling temperature capillary electrophoresis. Real-time PCR was used as a quantitative assay to determine the relative mtDNA copy number of 12 tumors studied, represented by two samples from each (*N* = 24); a high degree (75%) demonstrated tumor specimen heterogeneity. A grid of 96 spots isolated by laser capture microdissection demonstrated interfocal sample heterogeneity and increased the limit of detection. The spots demonstrated a wide range of mutant fractions from 0 to 100% mutant copies. The mitochondrial DNA copy number in the samples was determined by real-time PCR. No correlation between copy number and pathology-prognostic grade groups was observed. Somatic mitochondrial DNA point mutations represent traceable biomarkers demonstrating heterogeneity in primary prostate cancer. Mutations can be detected in areas before changes in tissue histopathology are evident to the pathologist.

## 1. Introduction

Prostate cancer (PCa) is the second most common cause of cancer and the sixth leading cause of cancer death among men worldwide [1]. In 2016, the incidence in Norway was 4983 new cases, and the mortality rate was 45.7 per 100 000 men [2]. Being strongly associated with age and low death rates in surveillance cohorts, the widespread use of radical treatment (surgery or radiation) with the subsequent reductions in quality of life calls for closer examination [3]. In prostate cancer, substantial interobserver variability represents a major limitation to the pathology grading system. Egevad et al. found that in a group of 337 uropathologists, only 56% agreement was achieved between expert consensus and participants' scores [4].

In the context of disease progression and treatment options, it is essential to increase the understanding of tumor characteristics. Hypothesizing that cancer is of clonal origin supports the idea of identifying cancer markers that enable cell lineage tracing. The growth of tumor cells is being accompanied by mutations in nuclear (nDNA) and mitochondrial genomes (mtDNA).

Numerous reports address the impact and importance of mitochondrial function in cancers, and mtDNA mutations are considered to be potential tumor biomarkers [5–9]. The 16,569 base pairs of mtDNA are prone to mutations, with an almost 10-fold higher mutation rate than the nuclear DNA [10, 11]. These frequent mtDNA mutations represent a collection of potential tumor markers. When compared to the nuclear genome, the mitochondrial genome in PCa shows a 55 times higher mutation rate [12]. An extensively observed somatic mitochondrial mutation would have occurred at an early stage in tumor development and spread through cell division. To effectively trace and measure its presence, we could identify areas being cancer-derived. Such labeling would be a quantitative observation not relying on subjective interpretation. This pilot study proposes an objective selection of microanatomical dissection of PCa tumors to be coupled with a quantitative analysis of mitochondrial point mutations. This procedure enables us to detect and trace somatic mtDNA mutations representing tumor lineages in human tumors [13]. The method used to quantify the mutant fractions was established in 1994 and has previously been used to analyze mutations in nuclear genes such as TP53, KRAS, NRAS, and BRAF as well as in the mitochondrial genome [13–22]. This pilot study aimed to examine the distribution of mtDNA mutation fractions in PCa in a linear tracing model and compare the maps of mitochondrial mutational heteroplasmy with the proposed prognostic grade group. The method used could complement future pathologic examination of human tumors. A series of 12 surgically removed prostate cancers were microanatomically examined. Grids of 2 × 96 microanatomical dissected samples from each prostate tumor were analyzed demonstrating the feasibility of this approach. This was validated when the distribution of mtDNA mutations was compared to the pathological tissue evaluation.

## 2. Materials and Methods

### 2.1. Specimen Samples

Surgical discharge data were collected from twelve patients undergoing Robot-Assisted Laparoscopic Prostatectomy (RALP) at Oslo University Hospital. The patients did not receive any neoadjuvant radiotherapy, androgen deprivation therapy, or chemotherapy before surgery. Written informed consent was required for participation:

 4 individuals from pathology ISUP grade group 1 (Gleason score ≤ 6)
 4 individuals from pathology ISUP grade group 3 (Gleason score 4 + 3 = 7b)
 4 individuals from pathology ISUP grade group 5 (Gleason score 9–10)
 2 samples from each surgical discharge *N* = 24 samples

Specimen samples were snap-frozen and stored at −70°C. All samples were anonymized with arbitrary numbers (REK approval no. 2018/111). From 12 whole-gland pathology specimens, two 6 mm cylinders were axially sampled from visible macroscopic tumor tissue. The cylinders were collected with an average intratumoral distance of 11.1 mm (range: 3 mm to 26 mm).

### 2.2. Tissue Sectioning

Samples were mounted on a cryostat sample holder with a water-soluble glycols and resins matrix (Tissue-Tek® OCT Compound, Sakura Finetek, USA). The sample holder temperature was set to −20°C and the knife temperature to −23°C. The cryostat (Leica CM1950) was set to cut five 50 *μ*m slices. These were collected in microcentrifuge tubes used for DNA extraction. Subsequently, a series of 12 *μ*m slices were mounted on LCM membranes (Leica frame slide, POL-membrane 0, 9 *μ*m, MicroDissect GmbH, Germany) and glass slides (Thermo Scientific, Gerhard Menzel, Braunschweig, Germany). Slides were stained with Giemsa as previously described [13].

### 2.3. DNA Extraction

Representative samples were obtained from 5 × 50 *μ*m portions of frozen tissue. DNA was extracted by NucleoSpin® Tissue protocol (Macherey-Nagel GmbH & Co., Düren, Germany) according to the manufacturer`s recommendations. Eluted DNA was stored at −20°C, to be analyzed later for somatic mitochondrial mutations. Additionally, 1 *μ*l DNA was extracted for the real-time PCR procedure.

### 2.4. First-Round PCR

Segments of mtDNA previously demonstrated to contain numerous somatic mutations [14] were amplified with mitochondrial-specific primers to avoid amplification of homologous regions in the nuclear DNA. Five sets of mitochondrial-specific primer pairs were used, resulting in amplification products between 714 and 928 base pairs in length. DNA amplification procedure was identical to that presented in [13, 14, 23].

### 2.5. Capillary Electrophoresis

All first-round amplification products were verified by capillary electrophoresis in MegaBACE 1000 DNA Analysis System (GE Healthcare Life Sciences, Pittsburgh, PA, USA). Samples were loaded into the capillaries from 96-well plates by electrokinetic injection at 161 V/cm for 20 seconds. The temperature of the capillary chamber was set to 27°C, and electrophoresis was carried out at a constant field of 145 V/cm.

### 2.6. Second-Round PCR

Templates for second-round PCR were 0.8 *μ*l of a 1 : 200 dilution (first-round PCR in H_2_O). The templates were dispensed into 96-well plates with a syringe dispenser (Hydra 96, Robbins Scientific, USA). To each well, 10 *μ*l reaction mixture was added. The components had a final concentration: 1 × ThermoPol Reaction Buffer with 2 mM MgSO4, 0.3 *μ*M primers without GC clamp, 0.15 *μ*M 1/2GC-tailed primer, 0.15 *μ*M, 6-carboxyfluorescein-GC-clamp, 500 *μ*M dNTP, 100 *μ*g Bovine Serum Albumin (Sigma-Aldrich, Oslo, Norway), and 0.75 U Cloned Pfu DNA polymerase. Plates were sealed with two strips of electrical tape (Clas Ohlson, Oslo, Norway). The temperature cycling was repeated 30 times: 94°C for 15 seconds, annealing temperature held for 30 seconds, and extension at 72°C for 60 seconds. Primers and annealing temperatures used to amplify fragments suitable for detecting mutations by CTCE are described in [14, 23].

### 2.7. Internal Standard

A fragment specific internal standard was created by amplifying PCR product (diluted 1 : 1000) and with the use of a forward primer containing a different fluorophore (ATTO532) and an unlabeled reverse primer. These were used as internal standards during electrophoresis and were injected into all capillaries in all runs before sample injection. The internal standard serves as a control of the separating temperature and as a marker for the mtDNA mutations.

### 2.8. Cycling Capillary Temperature Electrophoresis

After second-round DNA amplification, cycling temperature capillary electrophoresis (CTCE) was used to separate DNA variants in DNA from the LCM samples and to quantify mitochondrial mutant fractions. For a detailed description see [13, 14, 23].

### 2.9. Laser Capture Microdissection

A Leica DM6000 microscope was used to take images of tissue sections mounted on membranes. The software Leica laser microdissection V6.7.1.3952 was used to control the microscope when taking pictures or selecting areas for laser capture microdissection and cutting. A hardware modification was made to the collection unit allowing samples to be collected into strips of PCR caps (VWR, Oslo, Norway). Laser capture microdissection (LCM) subsamples or spots (2500 *μ*m^2^) were collected in 20 *μ*l collection solution (1 × ThermoPol Buffer with Proteinase K, 0.27 *μ*g/*μ*l). After cutting and collecting the selected areas by LCM, the strips (with collection liquid and tissue) were mounted onto a 96-well PCR plate (Axygen, VWR, Oslo, Norway). The plate was briefly centrifuged and incubated at 56°C for 30 minutes. Deactivation of Proteinase K was achieved by raising the temperature to 95°C for 1 minute. One microliter of the incubated solution was used as a template for the first-round PCR.

### 2.10. rtPCR Conditions

Real-time PCR was performed using the Bio-Rad CFX Connect Real-Time PCR Detection System. The PCR recipe was 2 × PerfeCTa SYBR Green SuperMix for iQ (QuantaBio, Beverly, MA, USA) and 0.2 *μ*M of each primer, for a final volume of 20 *μ*l. The PCR temperature cycling used was as follows: initial denaturing at 94°C for 4 minutes, followed by 45 cycles of denaturing at 94°C for 30 seconds, annealing at 60°C for 30 seconds, and extension at 72°C for 1 minute. Each sample was analyzed with 8 replicates for nDNA and 8 replicas for mtDNA targets. Primer information can be found elsewhere [24].

## 3. Results

**Figure 1:**
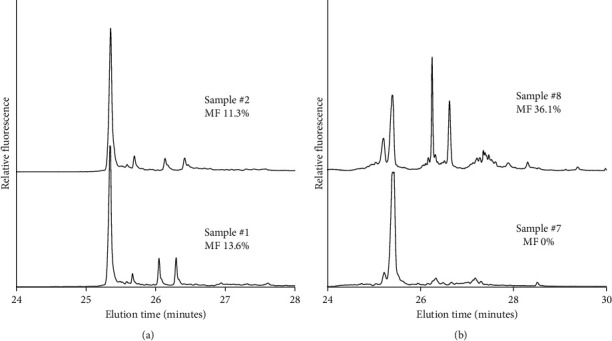
Electropherograms and peak separation as a result of cycling temperature capillary electrophoresis from initial tumor scan analysis. (a) Two samples representing the same prostate specimen, both demonstrating detected mtDNA mutations in prostate tumor 1. (b)Two samples representing the same prostate specimen, one of which demonstrating detected mtDNA mutations in prostate tumor 4.

DNA from 12 primary prostate cancers, each represented by two samples, was analyzed for hotspot mutations in the mtDNA. In the initial analysis, 75% (9/12) of the prostates had one or more mutations detected. Eighteen of the 24 samples (75%) were found positive for at least one mtDNA mutation (Table 1). Accordingly, nine sample pairs were subject to a second survey including LCM sampling and CTCE analysis.  Figure 1 displays peak separation as a result of cycling temperature capillary electrophoresis. Part A displays two representative electropherograms of mutated samples from the same prostate.  Figure 1(b) shows one mutated and one nonmutated sample from the same prostate specimen.

**Figure 2:**
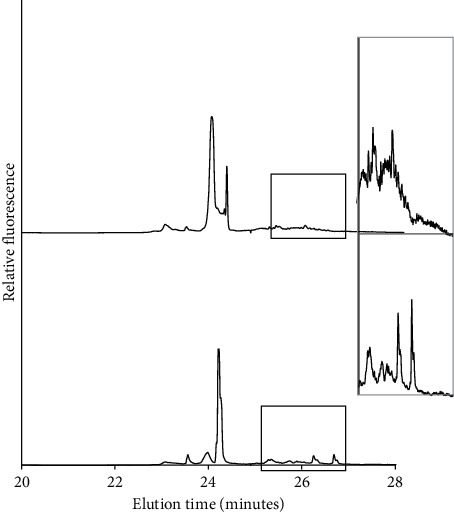
Electropherograms from initial tumor scan analysis. Two samples representing the same focus, one of which demonstrating detected mtDNA mutations in prostate tumor 3. The heteroduplex area is enlarged to illustrate 5% mutant fraction versus sample scored as nonmutated.

Figure 2 displays two electropherograms from the same tumor, representing different samples. The heteroduplex area is enlarged to illustrate 9.6% mutant fraction versus sample scored as nonmutated. The signal in sample #6 is given a mutant fraction 0% because the signal to noise ratio is below 2 (enlarged area). In three tumors, no mtDNA mutations were found in the initial scans (Table 1).

These samples were not further processed. This outcome suggests either no mtDNA mutations or an mtDNA mutation fraction below the detection limits of the assay. Consequently, nine sample pairs, a total of 18 samples, were subject to LCM. From each tumor sample, a grid of 96 circular spots (∼25000 *μ*m^2^) was dissected. Each sample spot was then analyzed by CTCE to quantify mitochondrial mutant fractions. Ninety-six LCM spots from each tumor, representing (2 × 9 × 96) 1728 samples, were further examined. The detection limit for each fragment analyzed is estimated at 1% [25]. This corresponds well with other studies using similar technique [17, 25–28]. Ten fragments where discordant with respect to mtDNA mutations in the initial scan. However, all mutations could be verified when the samples were subjected to LCM and mutation analysis (Table 1). This indicates that mtDNA mutation fraction is less than 1% in the initial tumor scan. When resampling with LCM, each “spot” contains a limited number of cells; this increases the chance of detecting mutant mtDNA, if cells carrying mutations are present (Table 1).

**Figure 3:**
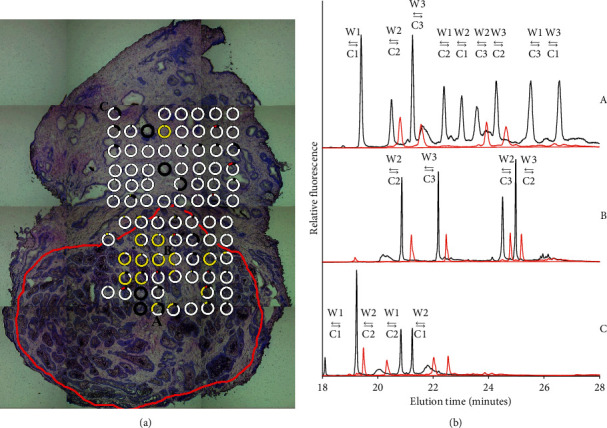
(a) Giemsa stained slice from prostate no. 3, sample 5, transposed mutant fractions obtained by focused laser capture microdissection in a grid of 8 × 12 spots. The red line encircles the tumor as defined by an experienced uropathologist. The circles in (a) show the LCM dissected areas and positions. White represents wild type or homoplasmy. The colors reflect the mutant fractions and their combinations. Corresponding electropherograms for the three circles labeled (A), (B), and (C) are reproduced in (b). (b) Electropherograms related to detected mtDNA mutations, their fractions, and combinations. The red signal represents a fragment specific internal standard used as a reference. Electropherogram (A) reflects mtDNA mutational combinations in the collected spot above the letter (A). Electropherogram (B) reflects mtDNA mutational combinations in the collected spot to the below-right of the letter (B). Electropherogram (C) reflects mtDNA mutational combinations in the collected spot to the below-right of the letter (C).

**Figure 4:**
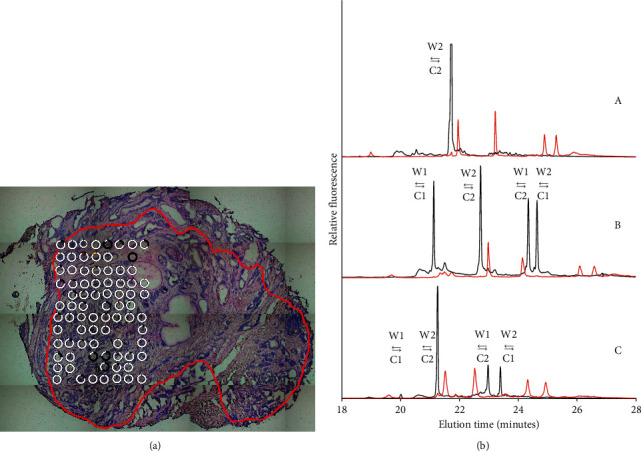
(a) Giemsa stained slice from prostate no. 3, sample 6, transposed mutant fractions obtained by focused laser capture microdissection in a grid of 8 × 12 spots. The red line encircles the tumor as defined by an experienced uropathologist. The circles in (a) show the LCM dissected areas and positions. White represents wild type or homoplasmy. The colors reflect the mutant fractions and their combinations. Corresponding electropherograms for the three circles labeled (A), (B), and (C) are reproduced in (b). (b) Electropherograms related to detected mtDNA mutations, their fractions, and combinations. The red signal represents a fragment specific internal standard used as a reference. Electropherogram (A) reflects mtDNA mutational combinations in the collected spot to the right of the letter (A). Electropherogram (B) reflects mtDNA mutational combinations in the collected spot to the right of the letter (B). Electropherogram (C) reflects mtDNA mutational combinations in the collected spot to the right of the letter (C).

Figures 3(a) and 4(a) exemplify two samples from the same prostate analyzed by the LCM and CTCE procedure. The red line encircles the tumor. The tumor borders were defined by an experienced uropathologist. The circles represent the LCM dissected areas and positions. The colors reflect the mutant fractions and their combination. The various fractions of mtDNA are quantified according to peak height in the electropherograms. The fragment specific internal standard was used as a reference for peak identification. Figures 3(b) and 4(b) depict electropherograms of LCM samples amplified and analyzed by CTCE (black). Additionally, the Watson–Crick strand combinations, as a result of PCR amplification with 1, 2, or 3 variants, are illustrated by arrows:

(a) 1 variant-maximum 1 peak
(b) 2 variants-maximum 4 peaks (Figure 1)
(c) 3 variants-maximum 9 peaks (Figure 3(b))

Internal standard (red) is amplified from a combination of variants illustrated as white and yellow circles. Sample 5 was analyzed and found mtDNA mutant positive in the initial sample scan. Sample 6 was analyzed and found mtDNA mutant negative in the initial sample scan.  Figure 4(a) depicts the detailed picture of mtDNA mutations in sample 6. The results illustrate detected mtDNA mutations at fractions as high as 90%. This indicates that the mutations have achieved homoplasmic or close to homoplastic levels. Even so, when analyzing the whole slice in the preliminary scan, no mtDNA mutations on that fragment were identified. This observation suggests that the mutation has reached levels close to homoplasmy although the cell lineage enclosing it was present at a total fraction less than 1% in the initial tumor sample. This assumption is supported by the large majority of white spots being noticed (i.e., wild type). All mutations identified in the initial scan were detected in the LCM samples. Additionally, LCM sampling and CTCE procedure identified mutation fractions in samples negative in the initial pairwise scan (Table 1).

**Figure 5:**
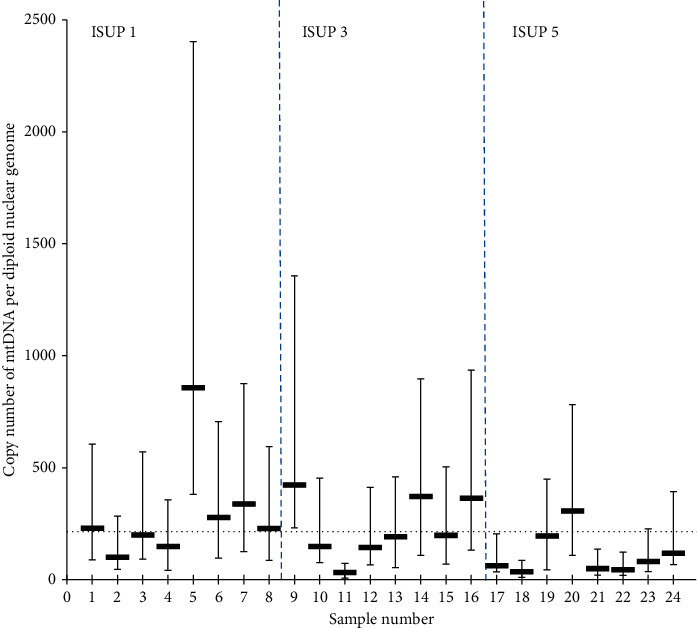
The average mtDNA copy number analyzed by real-time PCR on sample DNA. Data are plotted as average ±1 standard deviation from eight analytical replicates. Pairwise numbers, 1, 2, 3, and 4, etc., represent the same prostate, respectively. The horizontal line shows the mean copy number.

The mitochondrial DNA copy number from the initial tumor samples was determined by real-time PCR. The average copy number was 215 per cell (range 32–858). The copy number and range were in concordance with similar reports [8].  Figure 5 illustrates the mitochondrial copy number in the 24 samples and the corresponding ISUP group. The dotted horizontal line illustrates the average copy number value.

## 4. Discussion

This pilot study evaluates a method with the potential to systematically assess tumor heterogeneity in primary prostate cancer. It combines microanatomical sampling with mtDNA mutation analysis. The mtDNA of primary prostate cancer was scanned for mutations in known mutational hotspots [14]. The mtDNA hotspots were determined by CTCE when scanning 76% of the genome in 94 tumors of different origins [14]. The estimated detection limit of the method is in the order of 1% [13–22]. It separates DNA variants in a sieving matrix based on changes in the physical properties of the double helix due to mutations [29]. The peaks in the electropherograms represent amplified DNA from cells with and without the mutation. The method has been shown to be able to identify mutants within LCM captured samples [13]. The method proved to be a robust, cheap, and reproducible mutation detection pipeline to illustrate primary prostate cancer tissue heterogeneity. Given the number of LCM spots, each containing limited DNA for extraction, whole-genome sequencing was considered to be time-consuming, expensive, and technically impractical for this study.

In general, prostate cancers are adenocarcinomas of epithelial origin, with tissue heterogeneity made up by infiltrating nonmutated cells (either tumor or normal) which can alter the mutant fraction substantively (Figures 3(a) and 4(a)). When tracing mutations, the mutation detection method needs to be quantitative and have the ability to detect low mutant fractions. The use of focused sampling will increase the limit of detection. Laser capture microdissection is reported to be a powerful procedure for selective isolation of defined cell populations from heterogeneous tissue sections [30–32]. When mtDNA from the collected LCM samples was analyzed, the tumor heterogeneity was examined in a lineage tracing model assuming that mtDNA mutations are of monoclonal origin. The distribution of mtDNA mutations throughout a tumor sample can be hypothesized to reflect the pathology-prognostic grade group (ISUP) but requires a larger sample set than that used in this pilot study. When analyzing 76% of the mtDNA in 94 tumors of different tissues of origin, prostate cancer had an average of 3.2 mutated fragments per sample [14]. The sample number and average sequence coverage per genome were consistent with corresponding examinations [30, 31]. Heterogeneity in prostate cancer has been characterized by somatic mutations in nDNA [33]. Substantial experimental evidence suggests cancer initiation and progression are of clonal origin, i.e., originating from single cells with disruption of epigenetic control [34]. Detected somatic mitochondrial mutations would originate from a founding cell, to proliferate later through cell divisions to daughter cells.

The spot with an average size of 25000 *μ*m^2^ is a compromise between acceptable failure rate and resolution. Assuming an average cell diameter of 20 *μ*m, the theoretical upper bound is 80 cells/spot. In this study, the average cell count was 46 cells/spot. In a testicular tumor, the average cell number was reported to be 31 cells/spot [13]. Still, the multiple copies of mtDNA give enough templates for reproducible mutation analysis. The grid sampling and subsequent scanning of a limited quantity of cells increase the possibility of detecting areas with low mtDNA mutant fractions. These fractions were not detected in the initial scan (Figures 3(a) and 4(a)). In this manner, the dissimilarity in the initial analysis could not be reproduced, indicating a difference in the mutant fractions between the sample pairs. Thus, the assumed difference in heterogeneity across primary prostate tumor samples could not be verified. A sampling procedure limited to epithelial structures was believed to result in a possible loss of information. Accordingly, the LCM samples were collected objectively without considerations concerning tissue structure or staining. The results did not allow us to define where in the sample the mtDNA mutations could be detected, and the number of mtDNA mutations did not correlate with the ISUP grading; i.e., the pathology-prognostic grade group was not associated with the number of mtDNA mutations. In Figures 3(a) and 4(a), missing circles can be observed. This is due to loss of information in the steps of the protocol, which includes failure in LCM drop, DNA extraction, two rounds of PCR, and capillary electrophoresis. The LCM instrument depends on gravitation; consequently, spots <25000 *μ*m^2^ result in a higher capture failure rate. Increasing spot size dilutes the mutant fraction and decreases the resolution of tissue mutation distribution. Additionally, objective cutting could result in areas with little or no DNA, which would lead to signal failure.

Increased mtDNA copy numbers have been introduced as early molecular events during the initiation and progression of colorectal cancer [35, 36]. Zhou et al. indicated high mtDNA copy numbers in peripheral blood to be significantly associated with high Gleason score and advanced tumor stage [37]. When analyzing 384 primary prostate cancer tumors, the mtDNA copy number was reported to be strongly associated with Gleason score [8]. Accordingly, the relative mitochondrial DNA copy number and its distribution across the 3 pathology-prognostic grade groups were analyzed by real-time PCR. No correlation was observed between mtDNA copy number and increasing tumor aggressiveness as defined by pathologists. This outcome was observed with 8 technical replicas per sample. The analysis of rtPCR data was performed by using a robust regression analysis method [24]. The number of replicas in the order of 24 is required for confidence ratios below 1.5. When using 8 replicas, the average confidence ratio was 1.69. The confidence ratio was defined as the relative 95% confidence interval [24]. In primary prostate cancer, mtDNA molecular alterations indicate that tumor dynamics occurs in tissues adjacent and distant to histologically malignant glands without morphological indications of malignant transformation. Representing “field cancerification,” these early events may constitute discrete tissue remodeling to facilitate ensuing cancer progression [38]. Alternatively, the heterogeneity in somatic mtDNA mutations could represent tumors of common clonal origin but having acquired different mutational profiles. If so, this interfocal heterogeneity could indicate distinctive tumor driver mutations with individual cancer progression capacity. This observation is supported by studies characterizing prostate cancer metastases to be of monoclonal origin [39–41]. This consideration is not in conflict with the three-dimensional architecture of prostate cancer growth patterns. As recently demonstrated by Verhoef et al., the heterogeneity displayed in primary prostate cancer may represent growth pattern subgroups harboring tubular networks of tumor cells and serpentine contiguous epithelial proliferation. The two major architecturally different growth pattern subgroups representing interconnecting tubular structures consist of Gleason grade 3, poorly formed and fused Gleason grade 4, and cords of Gleason grade 5 [42]. Thus, the observed distribution and heterogeneity of mtDNA mutations could reflect a three-dimensional tumor matrix, illustrating the continuity of these growth pattern subgroups. These characterizations introduce considerable practical implications for today's snap-shot biopsy-based diagnosis. Future studies need to identify primary prostate cancer foci with metastatic potential. By integrating genomic and histopathological approaches, tumor characterization and subsequently clinical decision making will be refined.

## 5. Conclusion

Laser capture microdissection and CTCE-based point mutation assays effectively provide maps of mitochondrial DNA mutations demonstrating the heterogeneity in primary prostate cancer. Mitochondrial DNA copy numbers do not reflect the pathology-prognostic grade groups. The method was able to trace mutated cells within apparent normal tissue. If the concept of lineage tracing also could include the study of metastasis, this information could be of strategic relevance in the management of primary prostate cancer. Assuming that these markers are passenger mutations, we suggest that they have properties suitable for improved understanding of the lineage from the primary tumor to that of metastasis.

## Figures and Tables

**Table 1:** Prognostic grade groups (ISUP 1–5), consecutive prostate specimen, and the related sample number.

ISUP grade	Prostate #	Sample	Distance between samples (mm)	Mutant fraction (MF) in positive fragments			Mutation found by LCM when MF was 0% in initial analysis		
1	1	1	8	11.3%	20%				
		2		13.6%	9.3%				

1	2	3	26						
		4							

1	3	5	16	9.6%	0%			Yes	
		6		0%	12.6%		Yes		

1	4	7	21	20.1%	0%			Yes	
		8		16.1%	36.1%				

3	5	9	7	37.9%					
		10		13.5%					

3	6	11	9	12.7%	42.8%	2.2%			
		12		0%	0%	0%	Yes	Yes	Yes

3	7	13	10						
		14							

3	8	15	7	31%	36.9%	53.3%	Yes	Yes	
		16		0%	0%	30.8%	Yes		

5	9	17	4	0%					
		18		20%					

5	10	19	9						
		20							

5	11	21	3	6%	30%				
		22		4.8%	0%			Yes	

5	12	23	13	10%					
		24		40%					

Each specimen is represented by two samples. The sample number is used as a reference in all figures. The distance between the two collected samples from each index tumor was recorded with an average intratumoral distance of 11.1 mm (±6.8 mm). The samples from prostate 2 and 4 were collected from different tumor foci. Initial sample scan analysis was registered pairwise and 9/12 had one or more fragments with a detectable mutant fraction. This mutant fraction is reported as percentage. To increase the limit of detection, all nine sample pairs were subjected to LCM and CTCE analysis. All mutations detected in the initial scan were verified by this procedure. In the initial scan, tumor numbers 2, 7, and 10 did not demonstrate any positive mutant fractions in any fragments.

## Data Availability

All electropherograms, microscope pictures, or real-time PCR data are available from the corresponding author upon request.
